# Supplementary material for: COVID-19 Vaccine Acceptance among Liver Transplant Recipients
Source: Vaccines (Basel). 2021 Nov 11;9(11):1314. doi: 10.3390/vaccines9111314 (PMC8619490; doi:10.3390/vaccines9111314)
Supplement: Supplementary file 1 [file vaccines-09-01314-s001.zip › vaccines-1418290-supplementary.pdf]

Supplementary Material

S1. Questionnaire on COVID-19 vaccine hesitancy among liver transplant recipients. The translated adapted version of the validated questionnaire.

A) Personal and socio-demographic data

• Gender

□ M

□ F

• Age

___

•Nationality

□ ITA

□ UE

□ Not UE

•Place of birth

____

• Municipality (where You live and are being treated)

____

•Marital status

□ single

□ married

□ cohabiting

□ widower

□ married in second marriage

□ separated / divorced

• How many members are there in your household (including yourself)?

□ 1

□ 2

□ 3

□ 4

□> 4

• Do you have any children under the age of 10?

□ Yes

□ No

• What is your highest level of education obtained?

□ none

□ elementary school

□ middle school

□ high school

□ degree / post-graduate degree

• What is your current occupation?

□ student

□ employed part-time

□ busy full-time

□ precarious

□ unemployed

□ home-made

□ in layoffs

□ retired

□ wealthy without work

□ freelance

• What kind of profession do you have (or did you have)?

□ manager / entrepreneur / professional

□ employee / technical profession

□ manual / craft work

□ armed / police forces

• Do you work as health care provider?

□ Yes

□ No

B) Data relating to the disease

• Which is the reason of your liver transplantation?

□ Cirrhosis

□ Hepatocellular Carcinoma

□ Acute liver failure

• Are you currently being treated with? (more than one answer possible)

□ Tacrolimus

□ Ciclosporines

□ Corticosteorides

□ Azathioprine or 6-Mercaptopurine

□ Everloimus

□ Micophenolate

• How many years ago were you transplanted?

□ <5 years

□ 5-10 years

□> 10 years

• Do you follow the therapeutic recommendations?

□ Yes

□ No

□ most of the time

C) Lifestyles, behaviors, attitudes

• Do you smoke cigarettes / cigars?
□ Yes
□ No
□ Yes, electronic / non-combustion devices
□ Former smoker

• Do you consume alcohol?
□ Yes, often or in large quantities
□ Yes, minimal consumption
□ NO

• Do you think you have an active lifestyle (do regular physical activity, eg individual sport, running, walking, team games, gym)?
□ YES
□ No

• Are you vegetarian, vegan?
□ Yes
□ No

• Do you practice homeopathy, naturopathy or other alternative medicine?
□ Yes
□ No

• Do you undergo preventive diagnostic activities (pap-test, mammography, fecal occult blood test etc.)?
□ Yes
□ No

• What kind of attitude do you think you have towards vaccinations in general?
□ positive
□ partly positive, partly negative
□ negative

• Do you intend to get vaccinated again in the future?
□ Yes
□ No

• If you have children, do you intend to vaccinate your children in the future?
□ Yes, in full
□ Yes, in part
□ No

D) Knowledge and perceptions of vaccinations and vaccine preventable diseases

• Do you think vaccines are: (more than one response possible)
□ Effective
□ Safe
□ Necessary
□ Protective towards yourself
□ Protective towards the community
□ Free of adverse effects
□ With mild adverse effects
□ With significant adverse effects
□ in number and adequate vaccination sessions
□ too many
□ I don't know

• Do you believe that the diseases for which vaccines are planned are: (more than one response possible)

□ Not self-resolving
□ With long-term consequences (amputations, dementia, deafness, blindness, etc ...)

□ Potentially fatal
□ Easily contagious
□ Frequent
□ Paid by the whole community

• Do you believe that diseases for which vaccines are provided and which have disappeared from circulation, can return if vaccination is stopped?
□ Yes
□ No

• What do you think is the correct strategy (s) to prevent these diseases?
□ vaccination
□ alternative strategy to vaccination (healthy diet and physical activity, proper personal hygiene, food supplements, homeopathy, etc.)
□ vaccination and other strategies

• Can you distinguish between mandatory and recommended vaccinations?
□ Yes
□ No

E) Vaccination history

• Which of these vaccinations have you had in your life?
□ Tetanus
□ HBV (Hepatitis B)
□ HAV (Hepatitis A)
□ MPR (Measles-Mumps-Rubella)
□ Influence
□ Pneumococcus
□ HPV (Papilloma Virus)
□ Meningitis
□ Some (I don't know)
□ None
□ I don't remember

• Have you been vaccinated against one of the previous agents motivated by your inflammatory bowel disease or by the therapies you do?
□ Yes
□ No

F) Sources of information

• If you need information on vaccinations against the new Coronavirus responsible for COVID-19, who would you contact?

□ General Practitioner
□ Pediatrician
□ operator of the vaccination center
□ another health worker
□ pharmacist
□ relatives / friends / acquaintances / colleagues who work in healthcare
□ relatives / friends / acquaintances / colleagues who do not work in healthcare
□ scientific literature
□ mass media (TV, radio, newspapers)
□ internet
□ social networks

• Do you think you are informed about COVID-19 and its vaccination?
□ Yes
□ No

• If so, through which channel?
□ General Practitioner
□ Pediatrician
□ operator of the vaccination center
□ other health professionals
□ pharmacists
□ scientific literature
□ Ministry of Health (information campaigns, etc.)
□ relatives / friends / acquaintances / colleagues
□ mass media (TV, radio, newspapers)
□ websites
□ social networks

• Do you have a personal computer (PC), smartphone or tablet?
□ Yes
□ No

• Do you use the internet?
□ Yes
□ No

• If so, how often do you look for health information and health data?
□ Every day
□ A few times a week
□ At least once a month
□ Less than once a month
□ Never

G) Trust and Influences

• What do you think are the reasons that push the Ministry of Health and the Regional Health Department to strongly recommend vaccinations?
□ collective well-being / health
□ economic interests of pharmaceutical companies
□ other economic interests
□ both of the above

• Have you had any negative personal experiences (relating to yourself or your family, friends, acquaintances) with vaccines in the past?
□ Yes
□ No

• If so, which ones?
□ severe adverse reactions, such as allergy, shock
□ disabling diseases
□ autism
□ mild adverse reactions
□ perception of lack of protection

• Have you heard of negative experiences with vaccines on the internet in the past?
□ Yes
□ No

• If so, which ones?
□ severe adverse reactions, such as allergy, shock
□ disabling diseases
□ autism
□ mild adverse reactions (fever, pain, etc.)
□ perception of lack of protection

• Do you have more confidence in health professionals than in the mass media regarding information on vaccinations?
□ Yes
□ No

H) Liver transplantation and the new Coronavirus responsible for COVID-19

• Do you think that your Liver transplantation could expose you to a greater risk of contracting the new Coronavirus infection?
□ Yes
□ No

• Do you think that your Liver transplantation may be associated with a worse course of the disease?
□ Yes
□ No

• Do you think that the treatments you are taking for your Liver transplantation may increase the risk of contracting the new Coronavirus infection?
□ Yes
□ No

• Do you think that the treatments you are taking for your Liver transplantation may be associated with a worse course of COVID-19 disease?
□ Yes
□ No

I) Liver transplantation and vaccination against the new Coronavirus responsible for COVID-19

• Do you think that your Liver transplantation could expose you to a greater risk of having side effects from the new Coronavirus vaccine?
□ Yes
□ No

• Do you think that the treatments you are taking for your Liver transplantation could increase the possibility of having any unwanted effects from the new Coronavirus vaccine?
□ Yes
□ No

L) Intention to vaccinate against COVID-19

• If asked to get vaccinated against COVID-19 tomorrow, how would you respond?
□ Yes
□ No

• If answered yes, do you think your condition is a reason to access vaccination as a priority?
□ Yes
□ No

• If you answered no, for which of these reasons?
□ Fear of possible adverse reactions
□ I fear the speed at which the vaccine was developed

□ I think it is not effective
□ I am not afraid of the disease

• If you answered no, would you get vaccinated in the future when we have more data?
□ Yes
□ No

• If you answered no, was your choice influenced by having already had the disease (even if in an asymptomatic form diagnosed by antibody test)?
□ Yes
□ No

• If you answered no, was your choice influenced by your underlying chronic inflammatory disease?
□ Yes
□ No

Table S1. Socio-demographic of the total cohort of 711 patients.

Characteristic	(*n*=711)
Age, years, median (range)	63 (19-84)
Male gender, n (%)	502 (70.6%)
Years from liver transplant, median (range)	9 (0-35)
